# Activation of Transient Receptor Potential Vanilloid 4 Increases NMDA-Activated Current in Hippocampal Pyramidal Neurons

**DOI:** 10.3389/fncel.2013.00017

**Published:** 2013-03-04

**Authors:** Lin Li, Weijun Qu, Libin Zhou, Zihong Lu, Pinghui Jie, Lei Chen, Ling Chen

**Affiliations:** ^1^Department of Physiology, Nanjing Medical UniversityNanjing, China; ^2^State Key Laboratory of Reproductive Medicine, Nanjing Medical UniversityNanjing, China

**Keywords:** TRPV4, NMDA receptor, NR2B subunit, phosphorylation, excitotoxicity, cerebral ischemia

## Abstract

The glutamate excitotoxicity, mediated through *N*-methyl-d-aspartate receptors (NMDARs), plays an important role in cerebral ischemia injury. Transient receptor potential vanilloid 4 (TRPV4) can be activated by multiple stimuli that may happen during stroke. The present study evaluated the effect of TRPV4 activation on NMDA-activated current (*I*_NMDA_) and that of blocking TRPV4 on brain injury after focal cerebral ischemia in mice. We herein report that activation of TRPV4 by 4α-PDD and hypotonic stimulation increased *I*_NMDA_ in hippocampal CA1 pyramidal neurons, which was sensitive to TRPV4 antagonist HC-067047 and NMDAR antagonist AP-5, indicating that TRPV4 activation potentiates NMDAR response. In addition, the increase in *I*_NMDA_ by hypotonicity was sensitive to the antagonist of NMDAR NR2B subunit, but not of NR2A subunit. Furthermore, antagonists of calcium/calmodulin-dependent protein kinase II (CaMKII) significantly attenuated hypotonicity-induced increase in *I*_NMDA_, while antagonists of protein kinase C or casein kinase II had no such effect, indicating that phosphorylation of NR2B subunit by CaMKII is responsible for TRPV4-potentiated NMDAR response. Finally, we found that intracerebroventricular injection of HC-067047 after 60 min middle cerebral artery occlusion reduced the cerebral infarction with at least a 12 h efficacious time-window. These findings indicate that activation of TRPV4 increases NMDAR function, which may facilitate glutamate excitotoxicity. Closing TRPV4 may exert potent neuroprotection against cerebral ischemia injury through many mechanisms at least including the prevention of NMDAR-mediated glutamate excitotoxicity.

## Introduction

Stroke is a worldwide health problem leading to high rates of death and neurological disability in adults. The mechanisms underlying cerebral ischemia injury are complex, but the intracellular free calcium ([Ca^2+^]_i_) overload has been proved to play a vital role (Szydlowska and Tymianski, 2010). It is generally accepted that during cerebral ischemia, a large amount of glutamate accumulates in the synaptic cleft, which results in excessive calcium influx through *N*-methyl-d-aspartate receptors (NMDARs), to trigger and eventually induce cell death (Paschen, 1996). The glutamate excitotoxicity has long been recognized, however, treatment by directly targeting glutamate receptors has failed in clinical trials either because of intolerable side effects or lack of medical efficacy (Kemp and McKernan, 2002).

Apart from neuronal death, another critically important pathophysiological process in ischemic stroke is the formation of brain edema which includes the cytotoxic and vasogenic edema (Simard et al., 2007). The cytotoxic edema is the initial phase, which is caused by the energy failure leading to intracellular fluid accumulation. With ongoing of ischemia and reperfusion, cytotoxic edema becomes progressively evident and the secondary vasogenic edema eventually develops due to the disruption of blood-brain barrier (BBB). Brain edema is not only an important pathological process during cerebral ischemia, but also contributes to the adverse outcome (Marmarou, 2007; Simard et al., 2007). Therefore, prevention of brain edema or alleviation of brain edema-induced injury is critical for the treatment of cerebral ischemia.

Recently, there is accumulating evidence concerning the involvement of Transient Receptor Potential (TRP) channels, a potentially important calcium influx pathway, in cerebral ischemia injury. For example, TRP melastatin (TRPM) members, especially TRPM2 and TRPM7, are important contributors to the neuronal death following ischemia (Sun et al., 2009; Bai and Lipski, 2010). Besides TRPM channels, other TRP family members might also be responsible for ischemic neuronal injury. Among them, TRP vanilloid 4 (TRPV4) is attracting more and more attention because it can be activated by multiple stimuli including hypotonic stimulation, cell swelling, mild heat (>24-37°C), arachidonic acid (AA), and its metabolism epoxyeicosatrienoic acids (EETs; Plant and Strotmann, 2007), some of which may happen during stroke. TRPV4 is widely expressed in the central nervous system, including hippocampus, cortex, thalamus, and cerebellum etc. (Kauer and Gibson, 2009). During ischemia, the energy failure results in cytotoxic edema and membrane lipid metabolism disorder causing extra release of free AA (Westerberg et al., 1987; Marmarou, 2007; Simard et al., 2007). Therefore, it is very likely that TRPV4 is hyper-activated during ischemia, leading to an increase in calcium influx. Blockage of TRPV4 by lowering temperature may be responsible for the neuroprotection against oxygen-glucose deprivation-induced injury (Lipski et al., 2006). TRPV4 blockers GdCl_3_ and ruthenium red increase the viability of astrocytes exposed to oxidative stress (Bai and Lipski, 2010). But for the lack of selectivity of these blockers, the role of TRPV4 in cerebral ischemia injury remains to be determined. HC-067047 is recently described as a potent and specific TRPV4 antagonist. It is reported that HC-067047 can inhibit mouse TRPV4 activation by various stimuli, including heat, hypotonic stimulation, AA, 4α-PDD, etc. (Everaerts et al., 2010). The selectivity of HC-067047 makes it more promising and possible to explore the involvement of TRPV4 in cerebral ischemia.

Hippocampus is highly susceptible to ischemia among brain regions and pyramidal neurons of CA1 region are most sensitive to ischemia damage (Schmidt-Kastner and Freund, 1991). Concerning that the entry of Ca^2+^ through NMDAR is the major pathway leading to excitotoxic cell death associated with ischemia, the present study firstly tested the effect of TRPV4 activation on NMDA-activated current (*I*_NMDA_) in hippocampal CA1 pyramidal neurons and then explored the mechanisms underlying TRPV4-action. We also tested the effect of HC-067047 on brain infarction in focal cerebral ischemia model mice.

## Materials and Methods

### Animals

Male mice (ICR, Oriental Bio Service Inc., Nanjing) were used in the study. Care of animals conformed to standards established by the National Institutes of Health. All animal protocols were approved by the Nanjing Medical University Animal Care and Use Committee (ID: 20110628). All efforts were made to minimize animal suffering and to reduce the number of animals used.

### Slice preparation

Mice (3-week-old) were decapitated under deep anesthesia with ethyl ether. The brains were rapidly removed and the coronal brain slices (400 μm) were cut using a vibrating microtome (Microslicer DTK 1500, Dousaka EM Co, Kyoto, Japan) in ice-cold modified artificial cerebrospinal fluid (mACSF) composed of (in mM) NaCl 126, CaCl_2_ 1, KCl 2.5, MgCl_2_ 1, NaHCO_3_ 26, KH_2_PO_4_ 1.25, and d-glucose 20 oxygenated with a gas mixture of 95% O_2_/5% CO_2_. After 1 h recovery, hippocampal slices were transferred to a recording chamber.

### Electrophysiological recording

Whole-cell patch clamp recording were performed at room temperature (22–23°C). Hippocampal neurons were viewed with an upright microscope equipped with infrared-sensitive camera (DAGE-MTI, IR-1000). *I*_NMDA_ was recorded using an EPC-10 amplifier (HEKA Elektronik, Lambrecht/Pfalz, Germany), sampled at 10 kHz and filtered (Bessel) at 2.9 kHz. The capacitance and series resistance were compensated more than 90%. Data obtained from neurons in which uncompensated series resistance resulted in voltage-clamp errors >5 mV were not taken in further analysis. Liquid junction potentials were compensated before patching. When the external solution was changed, measurements of the changes in liquid junction potentials were <2 mV and were not corrected.

The resistance of glass pipettes was 4–5 MΩ filled with the pipette solution (in mM) CsCl 140, Tris-ATP 2, HEPES 10, EGTA 10 at pH 7.2. The slices were perfused continually with the oxygenated bath solution composed of (in mM) NaCl 74, CaCl_2_ 2.5, KCl 2.5, NaHCO_3_ 26, KH_2_PO_4_ 1.25, d-glucose 20, and d-mannitol 80 at osmolality of 300 mOsm/kg. When recording *I*_NMDA_, hippocampal pyramidal neurons were held at −60 mV. NMDA together with glycine (10 μM) were dissolved in bath solution and focally applied using a rapid drug delivery system directed toward the soma of recorded neurons. One micrometer strychnine, 10 μM bicuculline, 10 μM NBQX, and 0.1 μM tetrodotoxin (TTX) were added in bath solution to block glycine receptor, GABA_A_ receptor, AMPA receptor, and voltage-gated sodium channels, respectively. When recording 4α-PDD-evoked current, 10 μM 4α-PDD and 0.1 μM TTX were added in mASCF and a ramp protocol depolarizing from −80 to +80 mV over 700 ms was used. Hypotonic solution was obtained by adjusting the concentration of d-Mannitol. The osmolality was measured using the Advanced Micro Osmometer, model 3300 (Advanced instruments Inc., Norwood, MA, USA).

### Drug treatment

For intracerebroventricular (icv) implantation, mice (weighing 25–30 g) were anesthetized with chloral hydrate. A guide cannula (2.5 mm length, 23 gage) was implanted in the left lateral ventricle. HC-067047 stock solution was freshly diluted with 0.9% sodium chloride on the day of experiment. HC-067047 (10 μmol/2 μl/mouse) was injected with a stepper-motorized microsyringe (Stoelting, Wood Dale, IL, USA) at a rate of 0.5 ml/min. Control mice were given an equal volume of vehicle. HC-067047 was firstly injected 4 h (HC-4 h), 8 h (HC-8 h), and 12 h (HC-12 h) after middle cerebral artery occlusion (MCAO), respectively, and then injected every 8 h.

### Preparation of focal cerebral ischemia model

Three days after cannula implantation, focal cerebral ischemia was induced by MCAO as previously described (Mulcahy et al., 2003). Briefly, after mice were anesthetized, a poly-l-lysine (0.1%, weight/volume)-coated nylon monofilament thread (3/0 gage with the tip heat blunted to a diameter of 0.104 mm) was inserted through the external carotid artery and advanced into the internal carotid artery to occlude the origin of the middle cerebral artery (approximately 12 mm). Adequacy of vascular occlusion and reperfusion was monitored in the front parietal cortex of the occluded side with a multichannel laser Doppler flow-meter (Perimed PF5050, Sweden). Body and head temperatures were controlled at 37 ± 0.5°C with a thermostatically controlled heating pad. Arterial blood pressure and gases were monitored through a femoral catheter. After MCAO for 60 min, the filament was withdrawn for reperfusion. Sham-operated (sham-op) animals were treated identically, except that the MCAs were not occluded after neck incision.

### Infarction volume measurement

Brains were removed at 24 h post-MCAO, sectioned into five equidistant coronal slices (2-mm-thick), and incubated with a 2% 2,3,5-triphenyl-tetrazolium chloride (TTC) solution for 20 min to visualize infarct tissue, using an image analysis software (NIH-Image 3.12). Infarct volume was calculated as percentage of infarct area to the contralateral hemisphere area in each slice.

### Chemicals

4α-Phorbol-12,13-didecanoate (4α-PDD) was obtained from Calbiochem (San Diego, CA, USA) and TTX was obtained from Enzo life Science (Ann Arbor, MI, USA). Others, unless stated, all came from Sigma Chemical Company.

4α-PDD, HC-067047, d-Sphingosine, bisindolylmaleimide II (BIM), TBB, DRB and KN62, NBQX, and strychnine were prepared as stock solutions in DMSO. The final concentration of DMSO in the bath chamber or pipette solution was <0.1%. KN93, KN62, and d-Sphingosine were present in the pipette solution, while d(−)-2-Amino-5-phosphonopentanoic acid (AP-5), ifenprodil, PEAQX tetrasodium hydrate (NVP-AAM007), 4α-PDD, HC-067047, BIM, phorbol-12-myristate 13-acetate (PMA), TBB, DRB, NBQX, strychnine, bicuculline, and strychnine were added in bath solution.

### Data analysis

Data are expressed as means ± standard error and were analyzed with PulseFit (HEKA Elektronik) and Stata 7.0 software (STATA Corporation, USA). In the present study, after testing the effect of 4α-PDD and hypotonicity on *I*_NMDA_, 10 μM 4α-PDD was applied to the same neuron to test whether the neuron had TRPV4 receptors. All data came from the neurons where 4α-PDD-evoked current could be recorded. Before data analysis, both normality of the distribution and homogeneity of variance were assessed and data transformation (logarithmic or square root transformation) was considered whenever necessary. Paired or unpaired *t*-test and ANOVA followed by Bonferroni’s *post hoc* test were used for statistical analysis with the significance level set at *P* < 0.05. In dose-response curve, *I*_NMDA_ induced by different dose of NMDA was normalized to *I*_NMDA_ induced by 300 μM NMDA in isotonic solution (300 mOsm/kg) in the same neuron. The data were fitted to Logistic equation in which *I* = *I*_max_/[1 + (EC_50_/*C*)*^n^*], with *n* being Hill coefficient and EC_50_ being the concentration producing 50% maximal response. When exploring current-voltage relationship (*I*-*V* curve), *I*_NMDA_ induced at different holding potential was normalized to *I*_NMDA_ with the holding potential being −60 mV in isotonic solution in the same neuron.

## Results

### 4α-PDD increases *I*_NMDA_ in hippocampal CA1 pyramidal neurons

**Figure 1:**
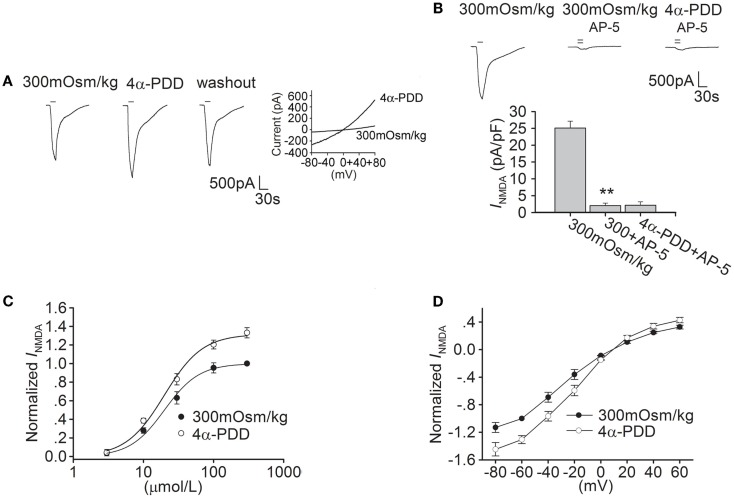
**4α-PDD increases *I*_NMDA_ in hippocampal CA1 pyramidal neurons**. **(A)** The typical recordings show that *I*_NMDA_ was increased from −1.93 to −2.52 nA after application of 4α-PDD for 5 min and the current recovered to −2.1 nA after washout. 4α-PDD-evoked current was recorded in the same neuron. **(B)** *I*_NMDA_ was reduced from -25.13 ± 2.01 to -2.05 ± 0.77 pA/pF by AP-5 (*n* = 6, paired *t*-test, *P* < 0.01). Note that in the presence of AP-5, the current was not changed by 4α-PDD. **P < 0.01 vs. 300 mOsm/kg **(C)** Dose-response curves for *I*_NMDA_ before and during 4α-PDD application. Each point represents the normalized current from 6 to 10 neurons. **(D)** *I*–*V* curve was shown in the presence of and absence of 4α-PDD.

As glutamate excitotoxicity is of great importance in cerebral ischemia injury, we firstly explored whether TRPV4 activation modulated NMDAR function. After application of 30 μM NMDA (for 20 s) and its co-agonist glycine onto hippocampal CA1 pyramidal neuron an inward current was recorded. This *I*_NMDA_ was blocked by the specific NMDAR antagonist AP-5 (50 μM), suggesting its mediation by NMDAR (Figure 1B). Here, it was found that *I*_NMDA_ was markedly increased by 31.6 ± 2.1% from -24.67 ± 1.04 to -32.33 ± 2.78 pA/pF after application of TRPV4 agonist 4α-PDD (10 μM) for 5 min (*n* = 10, paired *t*-test, *P* < 0.01). The increase in *I*_NMDA_ was reversible after 4α-PDD was washed out (Figure 1A). It was noted that in the presence of AP-5, 4α-PDD almost had no effect on the current (*n* = 6, paired *t*-test, *P* > 0.05; Figure 1B).

We then studied the effect of 4α-PDD on dose-response curve of *I*_NMDA_. EC_50_ and *n* values of dose-response curve were 19.91 ± 1.74 μM and 1.74 in the absence of 4α-PDD, respectively. After application of 4α-PDD, the maximal response to 300 μM NMDA was markedly increased (*n* = 6, paired *t*-test, *P* < 0.01), but EC_50_ (19.93 ± 1.67 μM) and *n* values (1.63) were almost unaffected (unpaired *t*-test, *P* > 0.05 in each case; Figure 1C).

We also performed experiments on current-voltage relationship of *I*_NMDA_. Application of 4α-PDD markedly increased *I*_NMDA_ at different voltages ranging from -80 to +60 mV. For example, when the holding potential was -80 mV, *I*_NMDA_ was significantly increased from -27.90 to -35.95 pA/pF (*n* = 8, paired *t*-test, *P* < 0.01). In *I*-*V* curve of *I*_NMDA_, the reversal potential was 9.61 ± 1.83 mV, which was not significantly different from the control (9.29 ± 1.58 mV; *n* = 8, paired *t*-test, *P* > 0.05). Besides this, we also compared the ratio of current at +60/-80 mV to find that the ration was not affected by 4α-PDD (control: -0.28; 4α-PDD: -0.29, *n* = 8, paired *t*-test, *P* > 0.05; Figure 1D).

### Hypotonic stimulation increases *I*_NMDA_ in hippocampal CA1 pyramidal neurons

**Figure 2:**
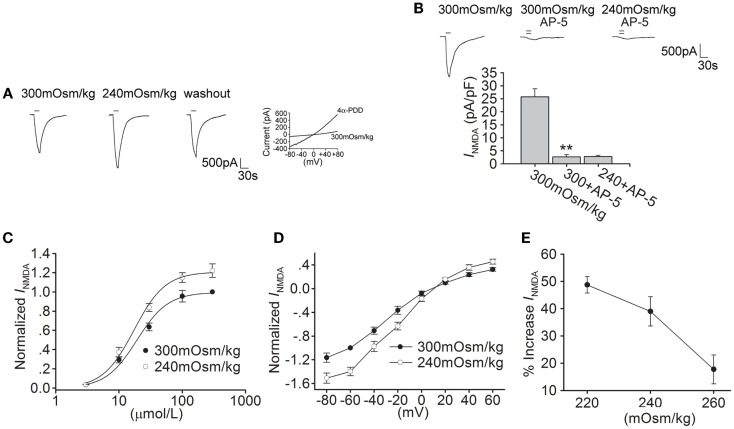
**Hypotonic stimulation increases *I*_NMDA_ in hippocampal CA1 pyramidal neurons**. **(A)** The typical recordings show that *I*_NMDA_ was increased from −1.73 to −2.42 nA when the extracellular isotonic solution (300 mOsm/kg) was changed to hypotonic solution (240 mOsm/kg) and the current recovered to −1.81 nA after washout. 4α-PDD-evoked current was recorded in the same neuron. **(B)** *I*_NMDA_ was reduced from -25.74 ± 3.12 to -2.67 ± 0.87 pA/pF by AP-5 (*n* = 6, paired *t*-test, *P* < 0.01). Note that in the presence of AP-5, the current was not changed by hypotonic stimulation. **P < 0.01 vs. 300 mOsm/kg. **(C)** Dose-response curves for *I*_NMDA_ in isotonic and hypotonic solution. Each point represents the normalized current from 7 to 17 hippocampal neurons. EC_50_ values were 19.23 ± 1.89 and 18.24 ± 1.07 μM, and *n* were 1.71 and 1.79 for isotonicity and hypotonicity, respectively. **(D)** *I*–*V* curves were shown in isotonic and hypotonic solution. **(E)** The plot shows that hypotonic stimuli exhibited more increase in *I*_NMDA_ with larger osmotic gradient.

As a cellular osmotic sensor, TRPV4 is sensitive to hypotonic stimuli. Here, we tested the effect of hypotonic stimulation on *I*_NMDA_. When the external solution was changed from isotonicity (300 mOsm/kg) to hypotonicity (240 mOsm/kg), *I*_NMDA_ was increased by 39.0 ± 5.4% from -25.01 ± 2.71 to -34.13 ± 2.82 pA/pF (*n* = 17, paired *t*-test, *P* < 0.05; Figure 2A). The increase in *I*_NMDA_ by hypotonic stimulation was largely reversible after hypotonicity was washed out for 5 min. The increase in *I*_NMDA_ was more evident with larger osmotic gradient (Figure 2E), and the following experiments were performed using hypotonic stimulation of 240 mOsm/kg which produced the significant increase in *I*_NMDA_.

As shown in Figure 2B, in the presence of AP-5, the current was almost unaffected by hypotonic stimulation (*n* = 6, paired *t*-test, *P* > 0.05). In hypotonic solution, the maximal *I*_NMDA_ was significantly increased, but EC_50_ and *n* values of dose-response curve were not markedly different in isotonic and hypotonic condition (paired *t*-test, *P* > 0.05 in each case; Figure 2C). Hypotonic stimulation increased *I*_NMDA_ at every voltage ranging from -80 to +60 mV, leaving the reversal potential (control: 9.52 ± 1.31 mV; 240 mOsm/kg: 10.02 ± 1.56 mV, *n* = 9, paired *t*-test, *P* > 0.05) and *I*(+60 mV)/*I*(-80 mV) ration unaffected (control: -0.28; 240 mOsm/kg: -0.29, *n* = 9, unpaired *t*-test, *P* > 0.05; Figure 2D). These results indicate that both 4α-PDD and hypotonic stimulation have similar effect on *I*_NMDA_.

### The specific TRPV4 antagonist HC-067047 attenuates 4α-PDD- and hypotonicity-induced increase in *I*_NMDA_

**Figure 3:**
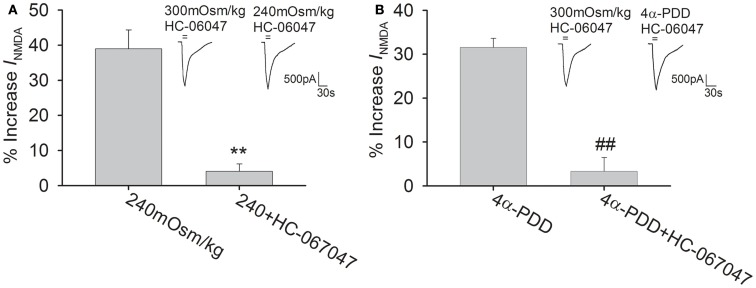
**TRPV4 antagonist blocks 4α-PDD- and hypotonicity-increased *I*_NMDA_**. **(A)** In the presence of HC-067047, *I*_NMDA_ was almost not changed by hypotonic stimulation and the increase in *I*_NMDA_ by hypotonicity was decreased from 39.0 ± 5.4% (*n* = 17) to 4.1 ± 2.2% (*n* = 21). **P < 0.01 vs. 240 mOsm/kg **(B)** Pre-application of HC-067047, the increase in *I*_NMDA_ by 4α-PDD was decreased from 31.6 ± 2.1% (*n* = 10) to 3.3 ± 3.1% (*n* = 18). ##*P* < 0.01 vs. 4α-PDD.

HC-067047 is a recently discovered specific TRPV4 antagonist (Everaerts et al., 2010). The present study showed that pre-application of HC-067047 (1 μM) markedly attenuated the increase in *I*_NMDA_ by both hypotonicity and 4α-PDD (unpaired *t*-tests, *P* < 0.01 in each case; Figure 3). Combined with the above results, it is suggested that activation of TRPV4 by either hypotonicity or 4α-PDD enhances *I*_NMDA_. The following experiments were performed in isotonic and hypotonic solution to explore the possible mechanisms underlying TRPV4-mediated increase in *I*_NMDA_.

### NR2B subunit is involved in hypotonicity-increased *I*_NMDA_

**Figure 4:**
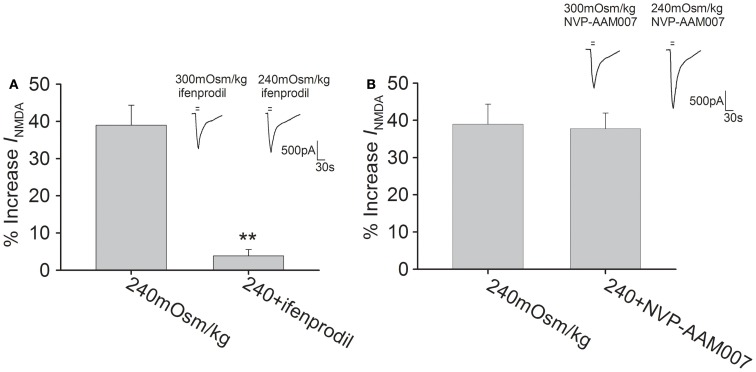
**NR2B subunit antagonist attenuates hypotonicity-increased *I*_NMDA_**. **(A)** In the presence of ifenprodil, the current was almost not changed by hypotonic stimulation and the increase in *I*_NMDA_ by hypotonicity was markedly attenuated from 39.0 ± 5.4% (*n* = 17) to 3.8 ± 1.8% (*n* = 18). **P < 0.01 vs. 240 mOsm/kg **(B)** Pre-application of NVP-AAM007, *I*_NMDA_ was increased 37.8 ± 4.2% (*n* = 14) by hypotonic stimulation, which was not different from the increase by hypotonicity alone.

Functional NMDAR is composed of both an NR1 subunit, which contains the glycine binding site, and an NR2 (A-D) subunit, which binds to glutamate. In the adult brain, both NR2A and NR2B subunits are prominent in the hippocampus (Laurie et al., 1997). In the presence of ifenprodil (10 μM), a specific NR2B subunit inhibitor, hypotonicity-induced increase in *I*_NMDA_ was markedly attenuated (*n* = 33, unpaired *t*-test, *P* < 0.01; Figure 4A). By contrast, pre-application of NVP-AAM007 (0.3 μM), a specific inhibitor of NR2A subunit, the increase in *I*_NMDA_ by hypotonicity was unaffected (*n* = 29, unpaired *t*-test, *P* > 0.05; Figure 4B).

### Calcium/calmodulin-dependent protein kinase II signaling pathways is involved in hypotonicity-increased *I*_NMDA_

**Figure 5:**
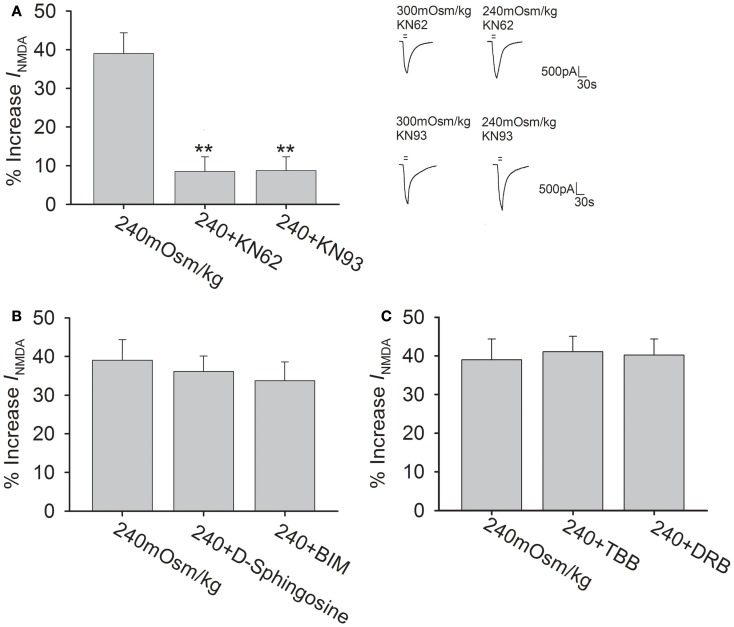
**CaMKII antagonists attenuate hypotonicity-increased *I*_NMDA._** **(A)** In the presence of KN62 or KN93, *I*_NMDA_ was almost not affected by hypotonic stimulation and the increase in *I*_NMDA_ by hypotonicity was markedly blocked by pre-application of KN62 or KN93. **P < 0.01 vs. 240 mOsm/kg. **(B)** *I*_NMDA_ was increased 36.2 ± 4.0% (*n* = 20) and 33.7 ± 4.9% (*n* = 12) by hypotonic stimulation in the presence of d-sphingosine or BIM, respectively. **(C)** Pre-incubation of TBB or DRB did not affect the increase in *I*_NMDA_ by hypotonicity.

The NMDAR subunits possess phosphorylation sites for protein kinases that can modulate the function of NMDAR (Chen and Roche, 2007). The following experiments were performed to test whether Calcium/calmodulin-dependent protein kinase II (CaMKII), protein kinase C (PKC), and casein kinase II (CKII) pathways were responsible for hypotonicity-increased *I*_NMDA_. As CaMKII plays an important role in phosphorylation of NMDAR, here we firstly evaluated the effect of CaMKII antagonists KN62 and KN93 on *I*_NMDA_ in isotonic solution. Pre-incubation of KN62 (5 μM) or KN93 (5 μM) decreased *I*_NMDA_ from -25.50 ± 1.15 to -21.01 ± 2.71 pA/pF (*n* = 7, paired *t*-test, *P* < 0.05) and from -25.08 ± 2.14 to -20.06 ± 1.56 pA/pF (*n* = 8, paired *t*-test, *P* < 0.05), respectively. As shown in Figure 5A, with KN62 or KN93 in the pipette solution, *I*_NMDA_ was increased 8.5 ± 3.8% (*n* = 15) and 8.7 ± 3.6% (*n* = 17) by hypotonicity, respectively, both of which were significantly different from hypotonicity-increased *I*_NMDA_ without antagonism of CaMKII (unpaired *t*-test, *P* < 0.01 in each case). This result suggests that CaMKII is responsible for the increase in *I*_NMDA_ caused by TRPV4 activation.

In isotonic solution, *I*_NMDA_ was increased from -24.42 ± 2.78 to -27.51 ± 0.84 pA/pF by PMA (agonist of PKC, 1 μM; *n* = 6, paired *t*-test, *P* < 0.05). After pre-application of PKC antagonists d-Sphingosine (20 μM) or BIM (1 μM), *I*_NMDA_ was decreased from -24.69 ± 0.94 to -21.63 ± 1.33 pA/pF (*n* = 9, paired *t*-test, *P* < 0.05) and from -25.04 ± 1.55 to -22.63 ± 2.64 pA/pF (*n* = 7, paired *t*-test, *P* < 0.05), respectively. Figure 5B shows that pre-incubation of d-Sphingosine or BIM did not affect the increase in *I*_NMDA_ by hypotonicity (unpaired *t*-test, *P* > 0.05 in each case). We also tested the role of CKII signaling pathway, for this pathway is reported to specially phosphorylate NR2B subunit. Here, it was found that application of CKII antagonist TBB (10 μM) or DRB (100 μM) decreased *I*_NMDA_ from -25.01 ± 5.95 to -18.19 ± 2.50 pA/pF (*n* = 7, paired *t*-test, *P* < 0.01), and from -24.94 ± 1.49 to -17.16 ± 1.57 pA/pF (*n* = 7, paired *t*-test, *P* < 0.01), respectively. Figure 5C shows that in the presence of TBB or DRB, *I*_NMDA_ was increased 41.1 ± 4.0% (*n* = 24) and 40.2 ± 4.7% (*n* = 10) by hypotonicity, respectively, both of which were similar to the increase in *I*_NMDA_ by hypotonicity alone (unpaired *t*-test, *P* > 0.05 in each case). These results indicate that neither PKC nor CKII signaling system is involved in TRPV4 activation-induced increased *I*_NMDA_.

### TRPV4 antagonist reduces brain damage after focal cerebral ischemia

**Figure 6:**
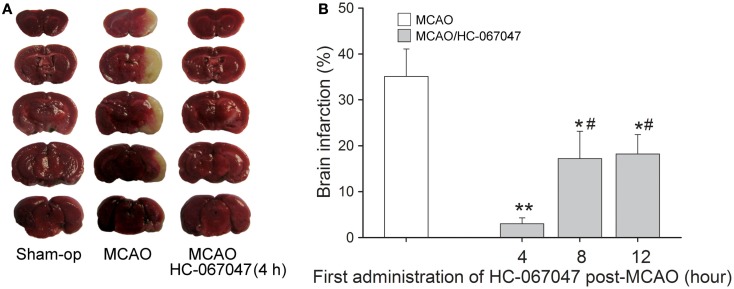
**TRPV4 antagonist reduces brain damage after focal cerebral ischemia**. **(A)** Representative photographs of cerebral infarction in MCAO mice treated with HC-067047. Note that treatment with HC-067047 (HC-4 h) after stroke significantly reduced the MCAO-induced cerebral infarction. **(B)** Time-window of neuroprotective effect of HC-067047. Bar graphs show the mean infarct volume percentage in MCAO mice that were treated with HC-067047 starting 4, 8, and 12 h post-MCAO. **P < 0.01 and *P < 0.05 vs. vehicle-treated MCAO mice, #*P* < 0.05 vs. HC-4 h.

The neuroprotection of blocking TRPV4 was tested *in vivo* using MCAO mice to induce focal cerebral ischemia. Figure 6A shows a representative experiment that the area of non-viable tissue, as indicated by pale color, was much smaller (3.0 ± 1.8%, *n* = 10) in the infracted hemisphere when mice were treated with HC-067047 (HC-4 h), compared with the mice treated with vehicle (35.3 ± 4.2%, *n* = 10; ANOVA followed by Bonferroni’s *post hoc* test, *P* < 0.01). Furthermore, HC-067047, when was firstly administered 8 h (HC-8 h, 17.1 ± 5.8%, *n* = 10) or 12 h (HC-12 h, 18.2 ± 4.1%, *n* = 10) post-MCAO, could markedly reduce the size of infarction (ANOVA followed by Bonferroni’s *post hoc* test, *P* < 0.05 in each case; Figure 6B). These results suggest that blockage of TRPV4 reduces MCAO-induced cerebral injury with an efficacious time-window of at least 12 h.

## Discussion

Cytotoxic edema is an important pathological process as well as a major cause of neuronal death in cerebral ischemia injury (Marmarou, 2007; Simard et al., 2007). Activation of TRPV4 is likely for its sensitivity to cell swelling, AA, and its metabolism EETs, which are always associated with cerebral ischemia. During ischemia, glutamate-induced intracellular calcium overload, which is mediated through NMDAR, plays an important role in neuronal injury. In this study, we found that application of 4α-PDD or hypotonic stimulation enhanced *I*_NMDA_ in hippocampal CA1 pyramidal neurons, which was markedly attenuated by the specific TRPV4 antagonist HC-067047 (Figure 3), indicating that activation of TRPV4 leads to the increase in *I*_NMDA_. Additionally, 4α-PDD- or hypotonicity-increased *I*_NMDA_ was blocked by NMDAR antagonist AP-5, indicating that TRPV4 potentiates NMDAR response (Figures 1B and 2B). It is known that NMDARs are calcium-permeable and the opening of these channels leads to further membrane depolarization and greater calcium influx, exacerbating intracellular calcium overload. Therefore, TRPV4-induced enhancement of NMDAR response may helps to facilitate glutamate-neurotoxicity during stroke.

The increase in *I*_NMDA_ probably results from an increase in NMDAR expression or enhanced NMDAR function. The present study mainly focused on the latter, for there is a report that NMDAR subunits expression is unaffected in TRPV4 knockout mice (Shibasaki et al., 2007). Although the maximal *I*_NMDA_ was increased, EC_50_ value in dose-response curve was not changed by either 4α-PDD or hypotonicity (Figures 1C and 2C), indicating that TRPV4-action is not dependent on increasing ligand binding affinity. Additionally, 4α-PDD and hypotonicity did not change the reversal voltage or *I*(+ 60 mV)/*I*(−80 mV) ration in *I*–*V* curve (Figures 1D and 2D), indicating that TRPV4-action is voltage-independent. It has been demonstrated that NR2B subunit is critical for a number of basic structural and functional attributes associated with NMDAR (Loftis and Janowsky, 2003). In this study, selective antagonism of NR2B but not NR2A subunit markedly blocked hypotonicity-induced increase in *I*_NMDA_ (Figure 4), suggesting the involvement of NR2B subunit in TRPV4-increased *I*_NMDA_. Increasing evidence supports the notion that the level of NR2B subunit phosphorylation is linked to the receptor activity, leading to the prolonged calcium influx. Our previous studies have reported that some intracellular signaling pathways are responsible for hypotonicity (TRPV4)-induced modulation on voltage-gated ion channels and TRPV1 receptor (Liu et al., 2007; Chen et al., 2008a,b, 2009; Li et al., 2011b). Here, CaMKII, PKC, and CKII signaling pathways were selected for they are responsible for phosphorylation of NR2B subunit (Chen and Roche, 2007). We found that the increase in *I*_NMDA_ by hypotonic stimulation was markedly attenuated by CaMKII, but not PKC or CKII antagonists (Figure 5). These results indicate that the phosphorylation of NR2B subunit by CaMKII is responsible for TRPV4-induced increase in *I*_NMDA_. It is known that activation of CaMKII is necessary before it phosphorylates NMDAR, which is dependence on Ca^2+^/CaM. TRPV4 activation increased *I*_NMDA_, which may facilitate calcium influx through NMDAR and exacerbate the increase of [Ca^2+^]_i_. Mg^2+^, which mainly exists inside of cell in normal condition, is very important for the activation of some kinases. The present recording solution was lack of Mg^2+^, suggesting that this recording condition may not be optimal condition for CaMKII activity. However, activation of CaMKII was still possible, which was caused by Mg^2+^ inside of neurons. Here, pre-application of CaMKII antagonists KN62 and KN93 inhibited *I*_NMDA_ in isotonic solution, which was consistent with the above deduction. It is reported that Ca^2+^/calmodulin-activated Ser-Thr kinase (CASK), which functions as an active protein kinase even without Mg^2+^ binding, may associated with CaMKII and modulate its phosphorylation state (Mukherjee et al., 2008). Whether CASK is involved CaMKII-induced modulation of NMDAR function remains unclear in the present study. On the other hand, the C terminus of TRPV4 contains a PDZ-binding-like motif that may contribute to the interaction of TRPV4 with PDZ-domain proteins (Garcia-Elias et al., 2008). Therefore, there may be other possibility that TRPV4 *per se* influences the activation of CaMKII.

**Figure A1:**
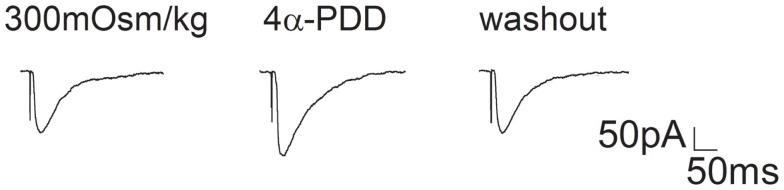
**Effect of 4α-PDD on EPSC in hippocampal slices**. EPSCs recorded from CA1 pyramidal neurons of hippocampal slices before, during and after 4α-PDD (10 μM) application. On the average, EPSC slope was enhanced 42.1 ± 5.0% by 4α-PDD (*n* = 8, paired *t*-test, *P* < 0.01). EPSC was evoked by stimulating Schaffer fibers through a constant-current pulse delivered by a Master-8 Stimulator (AMPI, Jerusalem, Israel). Hippocampal CA1 neurons were held at -70 mV and perfused with oxygenated bath solution composed of (in mM) NaCl 126, CaCl_2_ 1, KCl 2.5, MgCl_2_ 1, NaHCO_3_ 26, KH_2_PO_4_ 1.25, d-glucose 20, bicuculline 0.01, and TTX 0.1 μM. Pipette solution was composed of (in mM) Cs-gluconate 132.5, CsCl 17.5, MgCl_2_ 2, EGTA 0.5, HEPES 10, ATP 4, and QX-314 [*N*-(2,6-Dimethylphenylcarbamoylmethyl) triethylammonium bromide] 5 and pH was adjusted to 7.2 by CsOH.

Another important result of this study is that we firstly provide *in vivo* evidence that closing TRPV4 exerts potent neuroprotection against cerebral ischemic injury with at least a 12 h efficacious time-window (Figure 6). In fact, over-activation of TRPV4 may induce neuronal injury in cerebral ischemia through multiple mechanisms. In cultured hippocampal pyramidal neurons, application of glutamate induces more [Ca^2+^]_i_ increase at 37°C than at 25°C, which is more evident in wild type neurons than in TRPV4 knockout neurons. Moreover, this temperature-dependent [Ca^2+^]_i_ increase at 37°C was markedly inhibited by a NMDAR blocker in wild type but not in TRPV4 knockout neurons, which indicates that TRPV4 activation promotes NMDAR activation in hippocampal pyramidal neurons (Shibasaki et al., 2007). Consistently, the present study showed that activation of TRPV4 enhanced *I*_NMDA_ in hippocampal CA1 pyramidal neurons. On the other hand, activation of TRPV4 can depolarize the resting membrane potential (Shibasaki et al., 2007), which helps the release of presynaptic glutamate. Our experiment performed on the excitatory postsynaptic current (EPSC) also showed that TRPV4 agonist 4α-PDD increased EPSC in hippocampal slices (Figure A1 in Appendix), indicating that TRPV4 activation enhances synaptic transmission. Therefore, the enhancement of NMDAR response or/and increase in glutamate release is likely involved in TRPV4-mediated neuronal injury during stroke.

TRPV4 forms calcium-permeable, non-selective cation channels (Plant and Strotmann, 2007). Many studies including ours have reported that activation of TRPV4R causes an increase in intracellular calcium (Liu et al., 2007; Plant and Strotmann, 2007). Reactive oxygen species (ROS) and nitric oxide (NO) are important pathophysiological mediators of ischemia-induced toxicity (Loh et al., 2006). Recent studies performed in the urothelial cells, human coronary arterial endothelial cells, and lung macrophages have reported that activation of TRPV4 can stimulate the production of H_2_O_2_ and NO, which is mediated by TRPV4-induced increase in intracellular calcium (Donkó et al., 2010; Li et al., 2011a; Bubolz et al., 2012). Therefore, it is possible that during stroke, TRPV4 over-activation exacerbates ROS and NO production to induce neuronal injury.

It has recently been reported that TRPV4 and aquaporin-4 (AQP4) are co-expressed in astrocytic plasma membranes *in situ*, as well as in primary cultures and transfected cell lines (Benfenati et al., 2011). AQP4 plays an important role in keeping water balance in BBB and is involved in the formation of vasogenic brain edema (Zador et al., 2009). AQP4 and TRPV4 form a complex in the astrocytes that is essential for the brain’s volume homeostasis by acting as an osmosensor (Benfenati et al., 2011). Moreover, TRPV4 may participate in the pathogenic mechanisms of astroglial reactivity following ischemic insult because it is involved in ischemia-induced calcium entry in reactive astrocytes (Butenko et al., 2012). TRPV4 antagonists increase the viability of astrocytes in oxidative stress-induced cell damage (Bai and Lipski, 2010). The experiment performed on primary cultures of human respiratory epithelial cells shows that TRPV4 mediates calcium influx into human bronchial epithelia upon exposure to diesel exhaust particle, which leads to the activation of matrix metalloproteinase-1 (MMP-1; Li et al., 2011a). MMP-2 and MMP-9 are able to digest the endothelial basal lamina, resulting in opening of BBB. After cerebral ischemia, levels of MMP-2 and MMP-9 are increased, which plays an active role in the formation of brain edema and the secondary brain injury. More experiments will be needed to reveal a possible involvement of TRPV4 activation and MMPs activation in ischemia brain. Therefore, TRPV4 over-activation may also be responsible for the formation of vasogenic brain edema through facilitating AQP4 function or exacerbating the injury of astrocytes or/and basement membrane to increase the permeability of BBB.

In conclusion, this study shows that activation of TRPV4 potentiates NMDAR response, which may facilitate and prolong the glutamate excitotoxicity. Therefore, closing TRPV4 may effectively inhibit [Ca^2+^]_i_ overload and avoid the side effects through not directly inhibiting NMDAR. Ischemic injury is a complex insult, and treatment with a cocktail for multi-target is a more effective therapeutic strategy. The neuroprotection of TRPV4 antagonist exhibits long time-window (at least 12 h), which also indicates that the neuroprotective effect of closing TRPV4 may be mediated through multiple mechanisms. The present study suggests that TRPV4 is a promising novel target for treatment of ischemic stroke.

## Conflict of Interest Statement

The authors declare that the research was conducted in the absence of any commercial or financial relationships that could be construed as a potential conflict of interest.
