# Corrigendum: Activation of transient receptor potential vanilloid 4 increases NMDA-activated current in hippocampal pyramidal neurons

**DOI:** 10.3389/fncel.2014.00130

**Published:** 2014-05-19

**Authors:** Lin Li, Weijun Qu, Libin Zhou, Zihong Lu, Pinghui Jie, Lei Chen, Ling Chen

**Affiliations:** ^1^Department of Physiology, Nanjing Medical UniversityNanjing, China; ^2^State Key Laboratory of Reproductive Medicine, Department of Histology and Embryology, Nanjing Medical UniversityNanjing, China

**Keywords:** TRPV4, NMDA receptor, NR2B subunit, phosphorylation, excitotoxity, cerebral ischemia

The dosage of HC-067047 used in the article by Li et al. (2013) should be “10 μ M/2 μ l/mouse.” In addition, in appendix, the perfusion solution for recording EPSCs should be composed of (in mM): NaCl 126, CaCl_2_ 1, KCl 2.5, MgCl_2_ 1, NaHCO_3_ 26, KH_2_PO_4_ 1.25, D-glucose 20 and bicuculline 0.01.

## Conflict of interest statement

The authors declare that the research was conducted in the absence of any commercial or financial relationships that could be construed as a potential conflict of interest.
